# Impact of the ACOSOG Z0011 trial on surgical practice in Asian patients: trends in axillary surgery for breast cancer from a Korean Breast Cancer Registry analysis

**DOI:** 10.1186/s12957-022-02673-4

**Published:** 2022-06-13

**Authors:** Chihwan Cha, Eun Young Kim, Sung Yong Kim, Jai Min Ryu, Min Ho Park, Seokwon Lee, Young-jin Suh, Nayeon Choi, Hanpyo Hong, Hyung Suk Kim, Min Sung Chung, Korean Breast Cancer Society

**Affiliations:** 1 Department of Surgery, Hanyang University College of Medicine, 222, Wangsimni-ro, Seongdong-gu, 04764 Seoul, South Korea; 2 Department of Surgery, Kangbuk Samsung Hospital, Sungkyunkwan University School of Medicine, Seoul, South Korea; 3 Department of Surgery, Soonchunhyang University Cheonan Hospital, Cheonan, Chungnam South Korea; 4 Department of Surgery, Samsung Comprehensive Cancer Center, Samsung Medical Center, Sungkyunkwan University School of Medicine, Seoul, South Korea; 5 Department of Surgery, Chonnam National University Medical School & Hospital, Gwangju, South Korea; 6 Department of Surgery, Biomedical Research Institute, Pusan National University Hospital, Busan, South Korea; 7 Department of Surgery, The Catholic University of Korea St. Vincent’s Hospital, Suwon, South Korea; 8 Biostatistical Consulting and Research Lab, Medical Research Collaborating Center, Hanyang University, Seoul, South Korea; 9 Department of Surgery, Hanyang University Guri Hospital, Hanyang University College of Medicine, Gyeonggi-do, South Korea

**Keywords:** Breast cancer, Sentinel lymph node biopsy, Trends

## Abstract

**Background:**

Since the publication of the Z0011 trial, practice-changing clinical guidelines for breast surgery have been developed. Although recent studies confirmed the feasibility of the Z0011 strategy in Asian populations, there has been no study on the trends of axillary surgery in Asian cohort. This study aimed to investigate the time trend of axillary surgery for breast cancer from a Korean Breast Cancer Registry to understand the impact of the Z0011 trial in Asian patients.

**Methods:**

We collected prospectively constructed data from the nationwide Korean Breast Cancer Registry (KBCR). We identified patients who underwent sentinel node biopsy followed by breast-conserving surgery from 2011 to 2018 and were found to have pathological stage T1-2N1-3M0 disease. Regression analyses were performed to compare the downward trend of axillary lymph node dissection (ALND) in Korean cohort with that previously reported in a Dutch cohort.

**Results:**

From KBCR data, 7478 patients met the inclusion criteria. The proportion of ALND significantly decreased from 2011 (76.6%) to 2018 (47.5%). Multivariate analysis revealed that earlier years at diagnosis, larger tumor size, and lymphatic invasion were associated with a higher odds ratio of performing ALND. Compared to the Dutch cohort, the downward trend of ALND in Korea was significantly more gradual (annual percent change: 37.2 vs. 5.8%, *p* < 0.001).

**Conclusions:**

This study demonstrated a downward trend of ALND in Korean patients with breast cancer. However, the rate of decrease was significantly slower than that in the Dutch cohort.

## Introduction

The surgical approach to the axilla in patients with breast cancer has changed dramatically over the past two decades [1]. The results of recent randomized control trials have provided practice-changing recommendations for indications of axillary lymph node dissection (ALND) [2–5]. According to the American College of Surgeons Oncology Group Z0011 trial, patients who meet all the Z0011 selection criteria (cT1-2N0 disease with 1–2 positive sentinel nodes treated with breast-conserving surgery [BCS] followed by whole breast radiotherapy) do not gain benefit from completion ALND [6, 7]. Clinical practice recommendations such as the National Comprehensive Cancer Network guidelines and the American Society of Clinical Oncology guidelines were then modified to omit ALND for these patients [8, 9]. However, there have been wide variations in axillary management of patients with breast cancer among Western countries [10, 11]. It may be originated from that some physicians may not be aware of the recommendations or because they are reluctant to adhere to the guidelines. Indeed, some researchers have argued that the results of the Z0011 trial should be considered unreliable due to incomplete patient accrual and selection of a favorable subgroup consisting of postmenopausal women with small tumors [12–15]. Thus, it would be necessary to validate the Z0011 results in a larger cohort of patients with various conditions and then to modify, if necessary, and spread unified clinical guidelines to allow for evidence-based practices [10, 11].

There is similar uncertainty regarding an appropriate adoption of current surgical strategies, which have been established in Western countries, in Asian patients as breast cancers in Asian women have several characteristics different from those in Western women [16]. For instance, it has been reported that there are higher proportions of premenopausal patients and basal-like breast cancers in Asia than in Western countries [17, 18]. Therefore, while a few recent studies have demonstrated the feasibility of the Z0011 strategy in the Asian population, further validation studies would certainly benefit patients with breast cancer as well as breast surgeons in Asia [19, 20]. Meanwhile, there has been no study on the trends of axillary surgery in a large Asian cohort, and it is unclear whether or not surgeons in Asia have been slower to adopt the results of the Z0011 trial than those in Europe. Therefore, we aimed to analyze the trends of axillary surgery for breast cancer from a Korean Breast Cancer Registry to understand the impact of the Z0011 trial in Asian patients to assess the impact of the Z0011 trial over a period of time.

## Methods

### Korean Breast Cancer Registry (KBCR)

The KBCR is a web-based, prospectively maintained nationwide database managed by the Korean Breast Cancer Society. Since 1997, 102 institutions have participated in this registry. From the initial construction of the KBCR database, principal investigators from every institution have agreed on the principles and processes of utilizing this database for research. Essential registry items include patients’ unique national identification number, sex, age, type of surgery, and cancer stage according to the 7th edition of the American Joint Committee on Cancer classification. Moreover, data on biological characteristics (such as estrogen receptor [ER], human epidermal growth factor receptor 2 [HER2] status), and neoadjuvant and adjuvant treatment (such as radiotherapy, chemotherapy, and hormonal therapy) are collected. According to the guidelines of utilizing the KBCR database, this study was approved by the Institutional Review Board (IRB) of OOO University Hospital. The Korean Breast Cancer Society approved our research objective and our request to access data from July 2020.

**Fig. 1:**
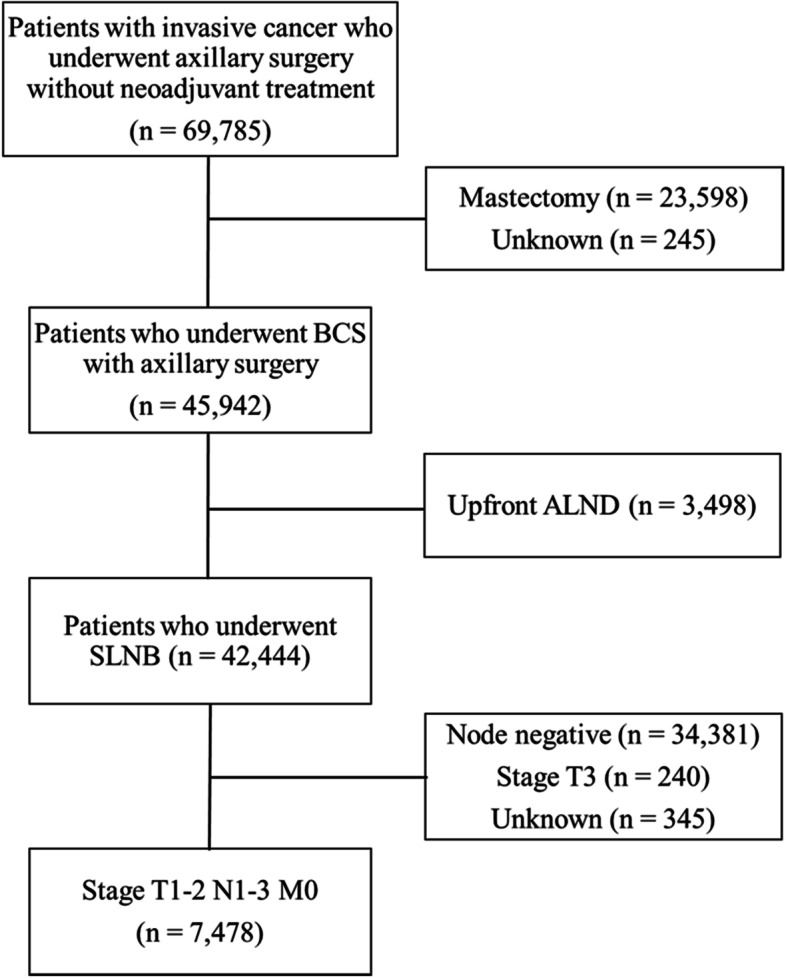
CONSORT diagram of the study population

Since information on the clinical stage was not available from the KBCR, we selected patients who would best meet the Z0011 criteria as follows. We identified patients who underwent sentinel lymph node biopsy (SLNB) as SLNB is performed in patients with cT1-2 N0 M0 disease. Of them, patients with pathological stage T1-2 N1-3 M0 disease who underwent BCS were finally selected (Fig. 1).

Data on the following variables were collected: age, year of surgery, histology, pathologic N stage, tumor grade, hormone receptor status, HER2 status, lymphatic invasion, number of nodes examined, number of positive nodes, and year-wise frequency of ALND from 2011 to 2018.

### Dutch population-based study: European cohort

The Dutch study population was described in detail in a previous study [21]. Briefly, data were obtained from their multidisciplinary nationwide registry of all diagnostic and treatment modalities performed in patients who had undergone surgical treatment for breast cancer from January 2011 to October 2015. Their study sample included 4959 patients diagnosed with cT1-2 N0 M0 invasive breast cancer and 1–2 positive sentinel lymph nodes after breast-conserving treatment. A positive sentinel node included micrometastases and macrometastases. Patients aged < 18 years, those who had received neoadjuvant systemic therapy, those with a prior history of breast surgery, and those whose data on axillary surgery was incomplete were excluded. Data from 85 Dutch hospitals were included in this study. We extracted the year-wise number and proportion of patients who underwent ALND between 2011 and 2015 from the published article and compared the downward trends of ALND in their population with those in our population.

### Study endpoints

The outcome of interest was trend in a surgical management of the axilla. Patients were divided into two groups based on the type of axillary surgery as follows: SLNB alone or SLNB + ALND. The primary endpoint was the year-wise percentage of patients who underwent ALND in Korean cohort between 2011 and 2018. The secondary endpoint was the difference in trends of axillary surgery between Korean and Dutch cohorts. In addition, we identified clinicopathological factors associated with the type of axillary surgery performed in Korean cohort.

### Statistical analyses

Descriptive analyses were used to analyze the trends in axillary surgery. Data are presented as frequencies and percentages (%) for categorical variables. Univariate associations between variables were examined using the chi-square test. Univariate and multivariate logistic regression analyses were performed to investigate the probability of performing ALND. Joinpoint regression analyses were used to compare the annual percent change (APC) of patients undergoing ALND between Korean and Dutch cohorts from 2011 to 2015. The statistical significance was set at *p* < 0.05. The statistical analyses were performed using SAS version 9.4 (SAS Institute Inc., Cary, North Carolina, USA). JOINPOINT Version 4.8.0.1 (Statistical Research and Applications Branch, National Cancer Institute) was used to fit the joinpoint regression model with an annual grid search.

## Results

### Characteristics of the Korean cohort

The clinicopathological characteristics of the 7478 Korean patients with T1-2 N1-3 M0 disease who had not received neoadjuvant chemotherapy and underwent BCS and SLNB from 2011 to 2018 are demonstrated in Table 1. The mean age was 51 years (range, 16–90 years), with 767 (10.3%) patients aged < 40 years and 2755 (36.8%) patients aged 40–49 years. Further, 44.4% of patients had pT2 lesions, with 487 (6.5%) patients having multifocal tumors. The histologic subtype was invasive ductal carcinoma (IDC) in 6232 (83.3%) cases. Approximately, 60% of patients had tumors with low histologic grade (I or II), and 2811 (37.6%) patients had a lymphatic invasion. ER-positive tumors were observed in 5534 (74.0%) cases, and HER2 overexpression was confirmed in 1047 (14.0%) cases. Patients with triple-negative breast cancer (TNBC) or HER2-positive subtype received more completion ALND than those with other subtypes (*p* = 0.021 in TNBC, *p* < 0.0001 in HER2-positive subtype). Among 6095 patients who had available data for radiotherapy, 5697 (93.5%) received radiotherapy and 398 (6.5%) did not.

**Table 1:** Clinicopathological characteristics of 7478 Korean patients who met the Z0011 criteria

	Total number (*n* = 7478)	SLNB + ALND (*n* = 4834)	SLNB alone (*n* = 2644)	*p* value^a^
Age at surgery (years)				
< 40	767 (10.3)	486 (10.1)	281 (10.6)	0.**738**
40–49	2755 (36.9)	1790 (37.0)	965 (36.6)	
50–59	2482 (33.2)	1612 (33.4)	870 (33.0)	
60–69	1060 (14.2)	690 (14.3)	370 (14.0)	
70–79	360 (4.8)	227 (4.7)	133 (5.0)	
> 80	48 (0.6)	27 (0.5)	21 (0.8)	
Unknown	6	2	4	
Year of surgery				
2011	1113	853 (76.6)	260 (23.4)	** < 0.0001**
2012	1252	909 (72.6)	343 (27.4)	
2013	1278	891 (69.7)	387 (30.3)	
2014	1241	784 (63.2)	457 (36.8)	
2015	801	487 (60.8)	314 (39.2)	
2016	727	394 (54.2)	333 (45.8)	
2017	784	382 (48.7)	402 (51.3)	
2018	282	134 (47.5)	148 (52.5)	
pT				
pT1	4156 (55.6)	2490 (51.5)	1666 (63.0)	** < 0.0001**
pT2	3322 (44.4)	2344 (48.5)	978 (37.0)	
Number of resected nodes (median, range)	12 (1–57)	16 (5–57)	5 (1–16)	** < 0.0001**
Number of positive lymph nodes (median, range)	1 (1–25)	2 (1–25)	1 (1–3)	** < 0.0001**
pN				
pN1	6381 (85.3)	3779 (78.2)	2602 (98.4)	** < 0.0001**
pN2	863 (11.5)	827 (17.1)	36 (1.4)	
pN3	234 (3.2)	228 (4.7)	6 (0.2)	
Multifocality				
Single lesion	5362 (91.7)	3407 (91.2)	1955 (92.5)	**0.094**
Multifocal lesion	487 (8.3)	328 (8.8)	159 (7.5)	
Unknown	1629	1099	530	
Histologic subtype				
IDC	6232 (83.3)	4067 (84.1)	2165 (81.9)	**0.013**
Other	1246 (16.7)	767 (15.9)	479 (18.1)	
Histologic grade				
I	915 (13.8)	600 (14.0)	315 (13.5)	** < 0.0001**
II	3605 (54.6)	2235 (52.3)	1370 (58.9)	
III	2085 (31.6)	1442 (33.7)	643 (27.6)	
Unknown	873	557	316	
Lymphatic invasion				
No	2899 (50.8)	1731 (46.9)	1168 (57.8)	** < 0.0001**
Yes	2811 (49.2)	1958 (53.1)	853 (42.2)	
Unknown	1768	1145	623	
ER status				
Negative	1212 (18.0)	843 (19.3)	369 (15.5)	** < 0.0001**
Positive	5534 (82.0)	3516 (80.7)	2018 (84.5)	
Unknown	732	475	257	
HER2 status				
Negative	5180 (83.2)	3299 (81.9)	1881 (85.5)	** < 0.001**
Positive	1047 (16.8)	728 (18.1)	319 (14.5)	
Unknown	1251	807	444	
ER/HER2 status				
ER + /HER2-	4450 (71.5)	2794 (69.4)	1656 (75.3)	** < 0.0001**
HER2 +	1047 (16.8)	728 (18.1)	319 (14.5)	
ER-/HER2-	729 (11.7)	505 (12.5)	224 (10.2)	
Unknown	1252	807	445	
TNBC				
Yes	670 (10.1)	460 (10.7)	210 (8.9)	**0.021**
No	5984 (89.9)	3839 (89.3)	2145 (91.1)	
Unknown	824	535	289	
Radiotherapy				
Yes	5697 (93.5)	3649 (93.7)	2048 (93.0)	**0.317**
No	398 (6.5)	245 (6.3)	153 (7.0)	
Unknown	1383	940	443	

*SLNB* Sentinel lymph node biopsy, *ALND* Axillary lymph node dissection, *pT* Pathologic tumor stage, *IDC* Invasive ductal carcinoma, *ER* Estrogen receptor, *HER2* Human epidermal growth factor receptor 2

^a^Categorical variables are tested by chi-square test and non-normally distributed numerical variables are tested by Wilcoxon rank-sum test

### Characteristics of the SLNB + ALND group in the Korean cohort

**Fig. 2:**
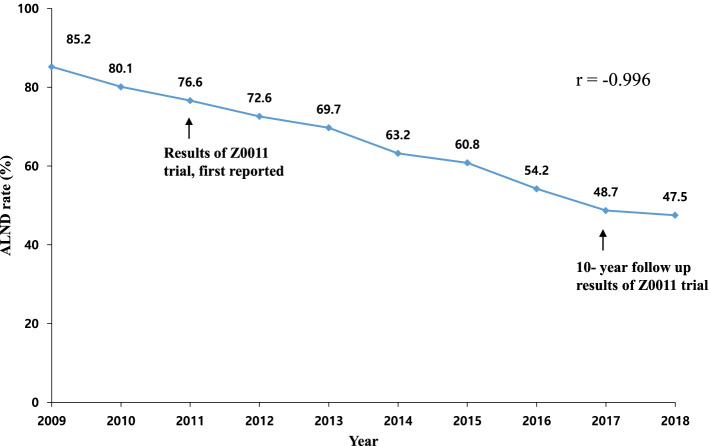
Rates of axillary lymph node dissection from 2009 to 2018 in Korean patients who met the Z0011 criteria. X-axis of graph, year of surgery; Y-axis of graph, proportions of patients who received ALND (%)

In 2009, prior to the publication of Z0011 results, 85.2% of patients who met the Z0011 criteria received ALND. The proportion of ALND gradually decreased from 2011 (76.6%) to 2018 (47.5%) (*p* < 0.001; Fig. 2). Joinpoint regression analysis indicated a significant reduction of 5.8% per year from 2011 to 2015 (*p* < 0.001). Univariate analysis for the performance of ALND according to the year of surgery demonstrated that the odds of undergoing ALND decreased over time (Table 2). A multivariate model adjusted for pathologic tumor stage, histologic subtype, histologic grade, lymphatic invasion, and ER/HER2 status revealed that larger tumor size, lymphatic invasion, ER-/HER2-subtype, and HER2-positive subtype were factors associated with a higher odds ratio of performing ALND (Table 3).

**Table 2:** Univariate analysis for the performance of axillary lymph node dissection according to the year of surgery

	Unadjusted OR	95% CI	*p* value
Year of surgery			
2011	Ref		
2012	0.808	0.670–0.973	0.025
2013	0.702	0.584–0.843	< 0.0001
2014	0.523	0.437–0.626	< 0.0001
2015	0.473	0.388–0.577	< 0.0001
2016	0.361	0.295–0.441	< 0.0001
2017	0.290	0.238–0.353	< 0.0001
2018	0.276	0.210–0.362	< 0.0001

**Table 3:** Univariate and multivariate analyses for the performance of axillary lymph node dissection in 7478 Korean patients who met the Z0011 criteria

Variables	Univariate analysis			Multivariate analysis^a^		
	Unadjusted OR	95% CI	*p* value	Adjusted OR	95% CI	*p* value
Age at surgery						
< 40	Ref					
40–49	1.072	0.908–1.267	0.410			
50–59	1.071	0.905–1.268	0.423			
60–69	1.078	0.888–1.309	0.446			
70–79	0.987	0.761–1.279	0.920			
> 80	0.743	0.413–1.340	0.324			
pT						
pT1	Ref			Ref		
pT2	1.604	1.455–1.767	< 0.0001	1.458	1.294–1.642	< 0.0001*
Multifocality						
Single lesion	Ref					
Multifocal lesion	1.184	0.972–1.442	0.094			
Histologic subtype						
IDC	1.173	1.035–1.330	0.013	1.279	0.992–1.649	0.058
Others	Ref			Ref		
Histologic grade						
I	Ref			Ref		
II	0.856	0.736–0.997	0.046	0.715	0.599–0.852	< 0.001*
III	1.177	0.998–1.389	0.053	0.872	0.707–1.076	0.202
Lymphatic invasion						
No	Ref			Ref		
Yes	1.549	1.388–1.728	< 0.0001	1.519	1.351–1.709	< 0.0001*
ER/HER2 status						
ER + /HER2-	Ref			Ref		
HER2 +	1.353	1.170–1.564	< 0.0001	1.263	1.066–1.497	0.007*
ER-/HER2-	1.336	1.129–1.582	0.001	1.246	1.015–1.529	0.036*

*pT* Pathologic tumor stage, *IDC* Invasive ductal carcinoma, *ER* Estrogen receptor, *HER2*, human epidermal growth factor receptor 2, *OR* Odds ratio, *CI* Confidence interval

^a^Adjusted for pathologic tumor stage, histologic subtype, histologic grade, lymphatic invasion, and ER/HER2 status

^*^P value < 0.05

### Comparison of trends on ALND between Korean and Dutch cohorts

**Fig. 3:**
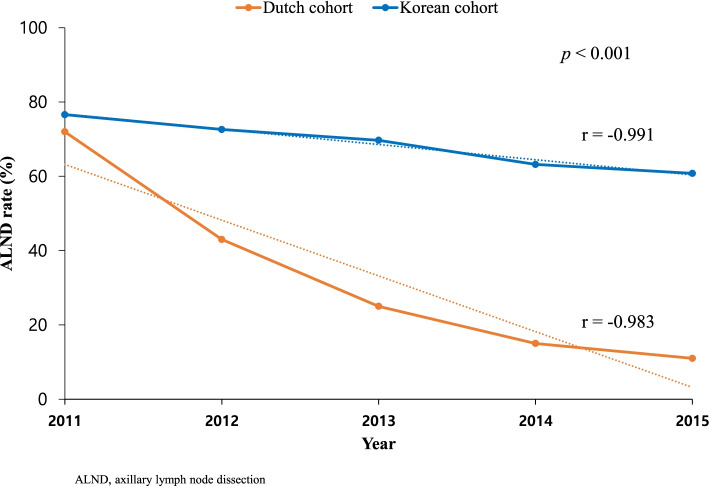
Downward trends of axillary lymph node dissection in the Korean and Dutch cohorts from 2011 to 2015. Blue line of graph: Korean cohort, orange line of graph: Dutch cohort.The dotted line is a trend line added

From 2011 to 2015, the downward trend of ALND in the Korean cohort was significantly more gradual than that in the Dutch cohort (APC 5.8 vs. 37.2%, *p* < 0.001, Fig. 3). Although 72% of Dutch patients and 76.6% of Korean patients underwent ALND in 2011, 11% Dutch patients, and 60.8% Korean patients underwent ALND in 2015 (Fig. 3).

## Discussion

The present study using prospectively constructed nationwide data from the KBCR demonstrated a downward trend of ALND in Korean patients with breast cancer who were eligible for the Z0011 criteria. However, compared to the Dutch cohort, the downward trend of ALND in Korea was significantly more gradual. Several characteristics, including tumor biology and demographics, vary between Asian and Western patients with breast cancer [17, 18]. Racial differences could affect the adoption of current surgical strategies established in Western countries. To the best of our knowledge, this is the first study to report the downward trend of axillary surgery in Asian patients with breast cancer and to compare the degrees of the downward trend of axillary surgery between Asian and Western countries.

Compared to the Z0011 participants, those in our study had larger tumor size, more frequent lymphatic invasion, and younger age. In the Z0011 cohort, approximately 30% of patients had T2 lesions, 28% of patients had lymphatic invasion, 25% of patients were < 50 years old, and 14.8% of patients were ER-/PR-subtype [6]. In contrast, in our study cohort, approximately 45% of patients had T2 lesions, 49% of patients had lymphatic invasion, 47% of patients were < 50 years old, and 10.1% of patients were TNBC. In the Dutch cohort, 37% of patients had T2 lesions, no information on lymphatic invasion was reported, and only 20% of patients were < 50 years old, similar to the Z0011 cohort [21]. A recent Japanese study by Kittaka et al. evaluated Japanese patients who met the Z0011 criteria and reported that their patients had larger tumors and more frequent lymphatic invasion than the Z0011 participants [22], which is consistent with our study findings. These differences in tumor biology and demographic characteristics between the Asian and Z0011 cohorts might underlie the difference in the trend of the surgical management of the axilla. We speculate that more unfavorable tumor characteristics in Asian women might be the reason why surgeons in Asia are less willing to omit ALND even in some patients who meet the Z0011 criteria. Our multivariate analysis revealed that larger tumor size and lymphatic invasion were associated with a higher odds ratio of performing ALND, indicating that surgeons preferred to perform completion ALND over SLNB alone in such cases.

Consistent with our findings, Liu et al. [19] reported that many surgeons in China preferred to perform completion ALND even in patients who met the Z0011 criteria. Although Chinese surgeons were aware of the Z0011 trial results, 42% of them still performed ALND in most cases considering that clinical situations in many areas of China were different from those in Western countries. For instance, many patients do not receive standard adjuvant treatment following surgery in suburban areas of China due to patient intention, financial issues, and medical conditions. Thus, some Chinese surgeons have argued that the Z0011 trial results may not be applicable in the clinical setting in China and that they prefer to choose radical surgery for any number of positive sentinel lymph nodes. In addition, some Chinese surgeons were uncertain whether the Western trial results could be applicable to the Chinese population due to differences in clinical characteristics between Asian and Western patients. Furthermore, there have been some debates on the potential selection bias related to the enrollment of patients in the Z0011 trial. Patients with a good prognosis alone were enrolled, and the trial was closed early with < 50% target accrual [23, 24]. The aforementioned explanations may also explain the downward trend of ALND in the Korean cohort that is not as fast as that in the Dutch cohort. As shown in Fig. 2, even after the 10-year follow-up results of the Z0011 trial were published in 2017, nearly half of the Korean patients (48.7%) still received ALND [7].

Several recent studies in large Asian cohorts have validated that the Z0011 strategy could be safely applied to Asian patients. Jung et al. [20] reported that ALND omission in the Asian cohort did not increase the risk of disease recurrence during a median follow-up period of 50 months. A prospective single-arm study by Kittaka et al. [22] showed that the 5-year cumulative rate of locoregional recurrences was only 1.3% in patients undergoing BCS with SLNB alone followed by radiotherapy. Another prospective single-arm study by Peng et al. [25] reported that only 1 patient (0.9%) had ipsilateral breast recurrence, and no regional recurrence occurred after a median follow-up of 29 months. Although prolonged follow-up is needed to determine the oncologic safety with regard to late recurrences, it might be feasible to apply the Z0011 strategy in Asian patients. Taken together, despite the differences in tumor biology and demographics along with clinical situations in Asian countries, more efforts are needed to spread the Z0011 strategy.

Previous studies have reported several factors associated with performing ALND in early breast cancer. According to recently published data from the European Society of Breast Cancer Specialists, the factors associated with a higher odds ratio of performing ALND in patients meeting the Z0011 criteria were the earlier years of surgery, younger age, larger tumor size, and a higher tumor grade [11]. In a Dutch population-based study, younger patients with invasive lobular subtype, those with a higher tumor grade, and those treated in a general non-teaching hospital underwent completion ALND more frequently. Our results similarly demonstrated that patients with poor prognostic tumor biology, such as large size and lymphatic invasion, underwent ALND more frequently than other patients. With regard to surgeons’ preferences, a survey study by Morrow et al. reported that surgeons in higher patient volume centers and those participating in a multidisciplinary tumor board had a lower propensity for performing ALND, suggesting the need for education targeting breast surgeons working in lower-volume centers [26].

Our study is limited by its retrospective design and lack of data on the clinical T and N stages. Instead, we postulated that patients with T1-2 N1-3 M0 disease who had undergone SLNB and BCS would best meet the Z0011 criteria. For this reason, about 14% of patients with pathologic N2-3 disease, who are not might be candidates for Z0011 trial, were included. It might be related with selection bias for study populations. Another limitation is the differences in the number of patients between study periods. Compared with the earlier years, the number of patients is smaller in the recent year, especially in 2018. It might be originated from the potential selection bias of registry data. Another limitation is that some pathologic parameters including gross extracapsular extension (ECE) were unavailable. As patients with gross ECE were excluded in the Z0011 trial, further studies including more pathologic data from other Asian countries would be warranted for validation of our results.

Despite these limitations, our study has a strength in analyzing large population-based nationwide data, including diverse surgeons’ preferences from multi-institutions. To our knowledge, this is the first study reporting real-world data on trends of axillary surgery in Asian patients with breast cancer. Furthermore, our study demonstrated that patients with TNBC or HER2-positive subtype who met the Z0011 criteria received more ALND than those with other subtypes. As TNBC or HER2-positive tumors are strong predictors of disease recurrence, a more aggressive surgical management for axilla seems to have been carried out [27, 28].

In summary, our study demonstrated a downward trend of ALND in Korean patients with breast cancer who met the Z0011 criteria. However, the rate of decrease was significantly slower than that in the Dutch cohort.

## Data Availability

The datasets generated during and/or analyzed during the current study are available from the corresponding author on reasonable request.

## Abbreviations

ALND: Axillary lymph node dissection
APC: Annual percent change
BCS: Breast-conserving surgery
KBCR: Korean Breast Cancer Registry
SLNB: Sentinel lymph node biopsy
